# A new generation of pPRIG-based retroviral vectors

**DOI:** 10.1186/1472-6750-7-85

**Published:** 2007-11-30

**Authors:** Olivier Albagli-Curiel, Yann Lécluse, Philippe Pognonec, Kim E Boulukos, Patrick Martin

**Affiliations:** 1 INSERM U790 and IFR54, Institut Gustave Roussy, PR1, 39 Rue Camille Desmoulins, 94805 Villejuif, France; 2 CNRS UMR 6548, Université de Nice, Parc Valrose, 06108 Nice, France

## Abstract

**Background:**

Retroviral vectors are valuable tools for gene transfer. Particularly convenient are IRES-containing retroviral vectors expressing both the protein of interest and a marker protein from a single bicistronic mRNA. This coupled expression increases the relevance of tracking and/or selection of transduced cells based on the detection of a marker protein. pAP2 is a retroviral vector containing eGFP downstream of a modified IRES element of EMCV origin, and a CMV enhancer-promoter instead of the U3 region of the 5'LTR, which increases its efficiency in transient transfection. However, pAP2 contains a limited multicloning site (MCS) and shows weak eGFP expression, which previously led us to engineer an improved version, termed pPRIG, harboring: i) the wild-type ECMV IRES sequence, thereby restoring its full activity; ii) an optimized MCS flanked by T7 and SP6 sequences; and iii) a HA tag encoding sequence 5' of the MCS (pPRIG HAa/b/c).

**Results:**

The convenience of pPRIG makes it a good basic vector to generate additional derivatives for an extended range of use. Here we present several novel pPRIG-based vectors (collectively referred to as PRIGs) in which : i) the HA tag sequence was inserted in the three reading frames 3' of the MCS (3'HA PRIGs); ii) a functional domain (ER, VP16 or KRAB) was inserted either 5' or 3' of the MCS (« modular » PRIGs); iii) eGFP was replaced by either eCFP, eYFP, mCherry or puro-R (« single color/resistance » PRIGs); and iv) mCherry, eYFP or eGFP was inserted 5' of the MCS of the IRES-eGFP, IRES-eCFP or IRES-Puro-R containing PRIGs, respectively (« dual color/selection » PRIGs). Additionally, some of these PRIGs were also constructed in a pMigR MSCV background which has been widely used in pluripotent cells.

**Conclusion:**

These novel vectors allow for straightforward detection of any expressed protein (3'HA PRIGs), for functional studies of chimeric proteins (« modular » PRIGs), for multiple transductions and fluorescence analyses of transduced cells (« single color/resistance » PRIGs), or for quantitative detection of studied proteins in independently identified/selected transduced cells (« dual color/selection » PRIGs). They maintain the original advantages of pPRIG and provide suitable tools for either transient or stable expression and functional studies in a large range of experimental settings.

## Background

Retroviral vectors are widely used to stably express any protein of interest. They potentially can transduce a large variety of primary or immortalized cells types, either *in vitro, ex vivo *or *in vivo*. Selection of transduced cells is generally accomplished by the co-expression of a fluorescent protein, a membrane protein or a gene encoding a drug-resistance. The more the expression of the two proteins correlates, the more the relevant cells in the selected population will be enriched. One way to obtain coupled expression in cells is to express both proteins from a single bicistronic mRNA: the 5' cistron encodes the protein of interest while translation of the 3' cistron is initiated by an IRES (Internal Ribosome Entry Site) element and gives rise to the selection protein. The pAP2 plasmid [1,2] belongs to this kind of retroviral vector since it contains eGFP as a selectable marker just downstream of the IRES element from the EMCV (encephalomyocarditis virus). Moreover, while many retroviral vectors harbor two complete LTR sequences, the U3 region of the pAP2 5' LTR has been replaced by the CMV immediate early enhancer/promoter. Hence pAP2 allows a potent CMV-driven expression upon transient transfection while retrotranscription (and integration) restores a complete, LTR-controlled, pro-viral sequence. However, despite this improvement, the pAP2 vector suffers from two major flaws: 1) its multicloning site (MCS) is limited; and 2) its IRES sequence is weak as a result of the creation of a HindIII site in its 3' terminus, which corresponds to two point mutations with respect to the original EMCV sequence. These mutations, though widely found in IRES containing vectors, destroy the last IRES ATG, normally used by the ECMV virus [2].

We thus constructed an improved derivative of pAP2 termed pPRIG (for plasmid Polylinker Retroviral IRES GFP) which keeps the advantages of pAP2 while eliminating its shortcomings. First, the pAP2 MCS had been replaced by a much more complete MCS which contains unique sites for twenty different restriction enzymes. This new MCS was optimized to allow directional cloning whatever the orientation of the cDNA since sites for compatible enzymes (e.g. BamHI and BglII) have been inserted quite symmetrically with respect to the center of the MCS. A T7 and a SP6 sequence were introduced at each extremity of the MCS to facilitate sequencing and allow *in vitro *transcription. Second, the IRES sequence had been changed to restore a wild type sequence, which enhances the translation of eGFP about 10 fold without affecting any other important parameters (e.g. viral titer or expression of the 5' cDNA). Finally, three pPRIG derivatives, termed pPRIG HAa/b/c, were also created. Each of these three derivatives contains an upstream HA tag encoding sequence in one reading frame with respect to that of the complete open ORF encoded by the pPRIG MCS [2].

Given these improvements, we reasoned that pPRIG is a very convenient basic vector to generate new useful retroviral tools. We provide here 14 novel pPRIG derivatives, collectively referred to as PRIGs, which keep the advantages of their founding member, but extend and facilitate the possibility of their uses to a wide range of studies.

## Results and Discussion

### 3'HA PRIGs

**Figure 1:**
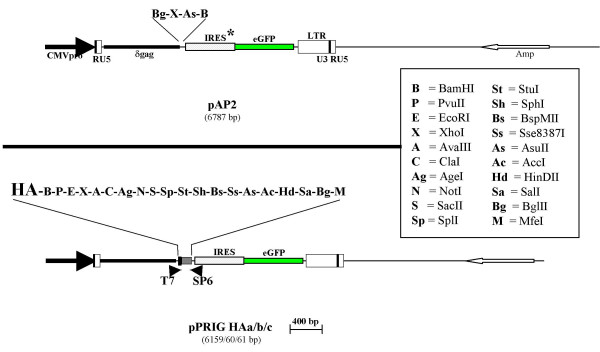
**Schematic representation of the pAP2 and pPRIG vectors**. The parental pAP2 retroviral vector harbors the CMV enhancer/promoter (CMVpro) in place of the U3 region of the 5' LTR and eGFP downstream of an ECMV-derived IRES containing two point mutations (hence indicated as IRES*) which strongly weakens its activity. The pPRIG HAa/b/c vectors improve pAP2 by: 1) replacing IRES* by the completely wild-type ECMV-derived IRES sequence (IRES), which restores its full activity; 2) inserting a much more complete multicloning site (MCS) and T7 and SP6 phage promoter on each side of the new MCS; and 3) adding a HA tag sequence in the three reading frames (a/b/c) 5' of the MCS. pPRIG HAa/b/c, as well as a pPRIG (without HA), were previously described and are used here to generate new vectors. For each vector, the unique sites of the MCS are indicated above the MCS.

In addition to the basic pPRIG vector (Fig. 1), we had previously described three derivatives bearing the HA tag coding sequence just upstream of the 5' site (BamHI) of the MCS [2]. The 5' HA coding sequence was inserted in the three reading frames relative to the MCS, giving the pPRIG HAa/b/c [2].

**Figure 2:**
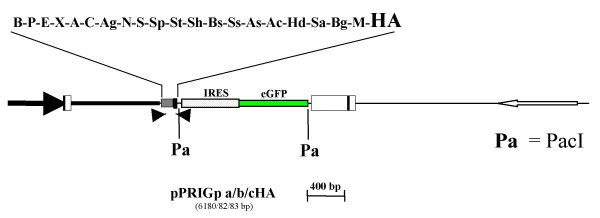
**Schematic representation of the HA3' PRIG vectors**. pPRIGp a/b/c HA are three pPRIG derivatives containing a HA tag sequence downstream of the MCS. Each of these derivatives harbors the HA sequence in one of the three reading frames relative to the MCS (hence pPRIG a/b/cHA). The reading frame is therefore open from and throughout the MCS to the stop codon located at the 3' end of the HA sequence. Moreover, the IRES-eGFP cassette is flanked by two PacI sites (Pa) a very rare cutter, hence pPRIG**p**a/b/c HA, allowing for accurate removal of the cassette. For each vector, the unique sites of the MCS are indicated above the MCS.

As a first step to design novel PRIG vectors, we created reciprocal constructs by inserting the HA coding sequence just downstream of the last restriction site (MfeI) of the basic pPRIG vector MCS in the three reading frames (pPRIGp a/b/cHA, Fig. 2). Importantly, although the 5' and 3' HA are symmetrically positioned relative to the MCS in our two sets of PRIG vectors, they are not fully equivalent. Indeed, given the scanning model ruling the choice of the initating ATG by the ribosome, the 5' HA is translated no matter what the sequence of the inserted cDNA is, while the 3'HA is optional since it is not translated if a stop codon is present in the inserted cDNA. A 3' tag may be especially useful if, for instance, the N-terminus of the protein of interest contains a cleavable signal peptide.

### "Modular" PRIGs

#### Expression of conditional alleles

**Figure 3:**
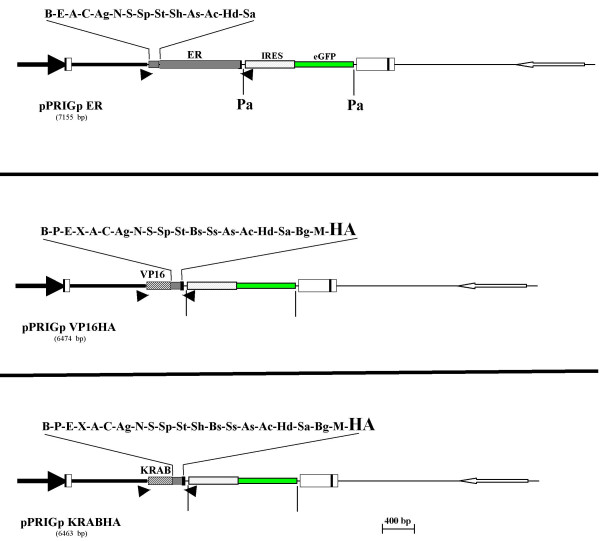
**Schematic representation of the « modular » PRIG vectors**. pPRIGp ER is a pPRIG derivative containing the C-terminal portion of the mouse estrogen receptor bearing the G525V mutation. This mutant does not bind estradiol but still binds the synthetic ligand hydroxytamoxifene. The ER sequence has been cloned at the 3' end of the MCS and the reading frame is thus open from and throughout the MCS to the stop codon ending the ER sequence. In contrast, in pPRIGp VP16HA and pPRIGp KRABHA, the sequence coding the functional module (VP16 transactivating domain from human herpes virus or KRAB transrepressing domain from human KOX1 protein) was cloned upstream of the MCS. The reading frame is thus open from the start codon of the module to the stop codon of the in-frame HA sequence 3' of the MCS. Pa: site for PacI. The two PacI sites of pPRIGp VP16HA and pPRIGp KRABHA are symbolized by vertical bars. For each vector, the unique sites of the MCS are indicated above the MCS.

The usual way to express a conditional version of many proteins is to fuse them to the hormone binding domain of the estrogen receptor. Consequently, the activity of the resulting chimeric protein is usually dependent upon the presence of ligand [3]. We thus cloned the hormone binding domain of the mouse ERα (ER, amino acids (aa) 281–599) downstream of the MCS of pPRIGp aHA, leading to pPRIGp ER (Fig. 3). This insertion therefore allows for the generation of X-ER chimera (Fig. 3). The presence of the G525R point mutation in the cloned ER sequence abrogates its binding to 17β-estradiol while keeping its full sensitivity to the synthetic ligand 4'hydroxytamoxifen (4OHT) [4]. Thus pPRIGp ER makes possible the culture of transduced cells expressing chimeric proteins in the presence of natural ER agonists without triggering basal activity [4]. Moreover, 4OHT does not activate the transactivating domain (termed AF2) of the cloned ER region [4,5], which therefore minimizes the risk of altering the function of the X protein moiety upon hormonal treatment. Although only one reading frame is available to construct X-ER chimera, we underline the presence of restriction sites for compatible enzymes in different reading frames within the PRIG MCS (e.g. StuI and HindII). Thus, the pPRIGp ER MCS provides different reading frames in a single sequence, provided that the 3'extremity of the X cDNA is clonable in one of the shifted compatible restriction sites. The same remark holds true for the fusions with any open sequence cloned in only one reading frame just upstream the PRIG MCS (KRAB, VP16, fluorescent protein), although in these cases the compatible restriction site has to be at the 5'end of the cDNA (see below).

#### Expression of transactivating or transrepressing chimera

The functional study of transcription factors frequently includes their fusion to potent transactivating or transrepressing domains. These fusions provide either dominant negative or dominant positive alleles, and help to determine whether a given DNA binding protein acts through transactivation, transrepression, or both [6,7]. To make possible such studies in PRIG vectors, we next constructed two derivatives by inserting the sequence encoding either the transactivating domain of the human herpes simplex virus VP16 protein [8] preceded by a short nuclear localization signal (KKKRK), or the N-terminal KRAB transrepressing domain of the human KOX1 protein [9] just upstream of the MCS of the pPRIG cHA, generating pPRIGp VP16HA or pPRIGp KRABHA, respectively (Fig. 3). Both domains are relatively short (corresponding to aa 413–490 or 1–89 of the original protein, respectively), highly potent, transferable and widely used to generate chimeric transregulatory proteins [10-12]. The unique restriction sites of the MCS are entirely preserved, with the exception of SphI in pPRIGp VP16HA (Fig. 3). These two modular PRIG vectors provides an in-frame 3'HA coding sequence (Fig. 3) and are therefore engineered to create KRAB-X or VP16-X chimeras with or without a C-terminal HA tag depending upon the absence or presence of a stop codon in the inserted cDNA.

### « Single-color/resistance » PRIGs

#### "single color" PRIGs

**Figure 4:**
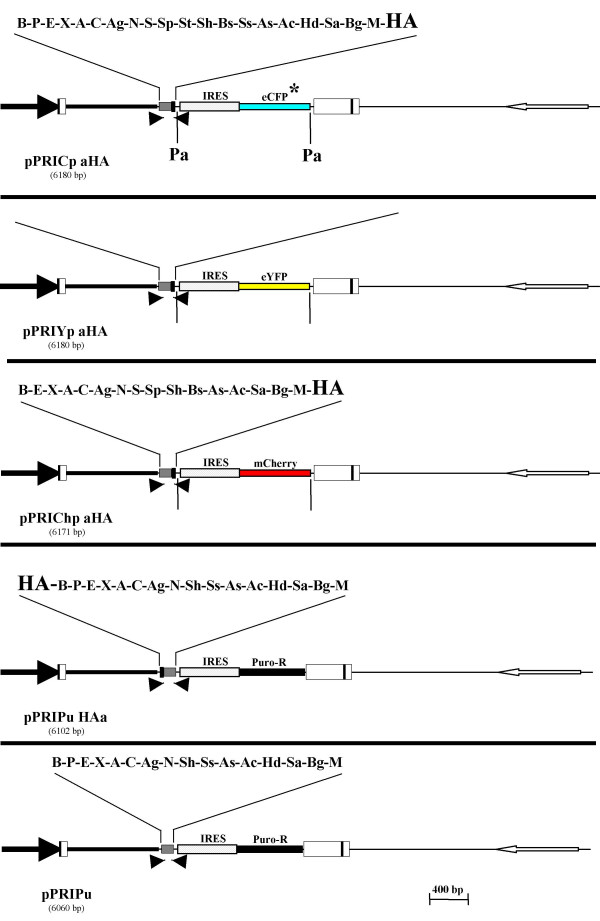
**Schematic representation of the « single color/resistance » PRIG vectors**. pPRICp aHA, pPRIYp aHA and pPRIChp aHA and pPRIPu vectors are pPRIG derivatives in which the eGFP sequence from the pPRIG has been replaced by eCFP*, eYFP, mCherry or Puro-R coding sequence, respectively. eCFP* is a derivative of eCFP bearing the H148D mutation which enhances its brightness. pPRICp aHA, pPRIYp aHA and pPRIChp aHA contain an in-frame 3' HA sequence (frame « a ») with respect to the MCS. They also contain the two PacI sites flanking the IRES-XFP cassette (shown as vertical bars in pPRIYp aHA and pPRIChp aHA). By contrast, pPRIPu is devoid of both HA sequence and PacI sites. For each vector, the unique sites of the MCS are indicated above the MCS. Unique sites of the pPRIYp aHA MCS are not listed since they are the same ones as in the immediately above vector.

Though eGFP is widely used to track transduced or, more generally, genetically modified cells, it has also several yellow or blue shifted suitable derivatives [13]. Moreover, highly performant red fluorescent proteins were generated by directed evolution of mRFP, which is itself a monomeric derivative of the DsRed protein [13,14]. We reasoned that PRIGs harboring one of these fluorescent proteins instead of eGFP may extend the number of issues amenable by these vectors. Hence, three other PRIGs were generated in which the eGFP of pPRIGp aHA has been replaced by either eCFP containing the H148D mutation, eYFP or mCherry, generating pPRICp aHA, pPRIYp aHA and pPRIChp aHA, respectively (Fig. 4). eCFP is a blue shifted eGFP derivative [14]. Introduction of the H148D mutation (eCFPH148D, thereafter termed eCFP*) increases two-fold its quantum yield and brightness without affecting its spectral properties [15]. eYFP is a bright and widely used yellow shifted eGFP derivative. mCherry is a monomeric ("m") red fluorescent protein which displays very similar spectral properties, but improved photostability, compared with its ancestor, mRFP [13]. Moreover, we also replaced eGFP by eCFP* in pPRIGp bHA, leading to pPRICp bHA, which is required for another derivative (see below). Each of these fluorescent proteins is compatible (*i.e*. well spectrally separated) with a distinct set of fluorochromes. Thus, these three novel single-color PRIGs provide convenient tools to combine an identification (or sorting) of transduced cells together with other fluorescent-based analyses (e.g. expression of markers). Moreover, eCFP* and eYFP can be well separated spectrally from each other, and mCherry is readily distinguishable from eCFP*, eGFP and even from eYFP because of its red-shifted spectral properties as compared with DsRed [13,14,16]. The four single color PRIGs are therefore also suitable for performing double or even triple transductions, which may be required in many situations to induce biological changes [17].

#### « Drug-selectable » PRIGs

In addition, since FACS-based selection of transduced cells is efficient and rapid but requires expensive materials, we also constructed two other PRIGs that contain the puro-resistance gene instead of eGFP in either pPRIG or pPRIG HAa, leading to pPRIPu or pPRIPu HAa, respectively (Fig. 4). Puromycin inhibits translation elongation, and is a relatively cheap drug for selecting transduced cells and acts rapidly even at very low concentrations. pPRIPu and pPRIPu HA vectors can also be used with any of the fluorescent protein expressing vectors (or any combination of them) further extending the possibility of multiple transductions with PRIGs.

### « Dual color/selection » PRIGs

#### « Double color » PRIGs

**Figure 5:**
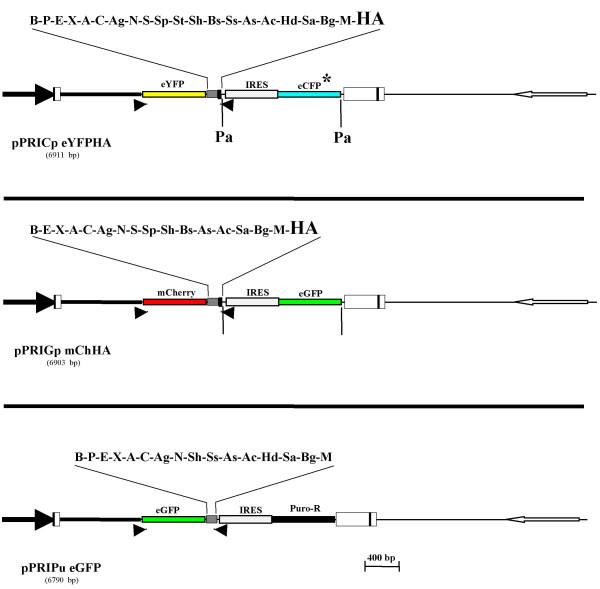
**Schematic representation of the « dual color/selection » PRIG vectors**. pPRICp eYFPHA is a pPRICp aHA derivative in which the eYFP sequence has been cloned upstream of the MCS. pPRIGp mChHA is a pPRIGp aHA derivative in which mCherry sequence has been cloned upstream of the MCS. pPRIPu eGFP is a pPRIPu derivative in which the eGFP sequence has been cloned upstream of the MCS. The reading frame is open from the start codon of the upstream fluorescent protein to the stop codon of the HA sequence (for pPRICp eYFPHA and pPRIGp mChHA) or to the in-frame stop codon 3' to the MCS (for pPRIPu eGFP). pPRICp eYFPHA and pPRIGp mChHA, but not pPRIPu eGFP, harbor the two flanking PacI sites. For each vector, the unique sites of the MCS are indicated above the MCS.

Cells transduced with any of the PRIG vectors described above express a bicistronic cDNA, the IRES-controlled translation of the 3' cistron giving rise to a fluorescent or selectable protein. Although the expression of the IRES-controlled protein usually correlates with that encoded by the 5' cistron, a direct detection of the protein of interest in transduced cells can be valuable. The best way to make this direct detection easy and quantitative is to express chimeric fluorescent proteins from the 5' cistron, keeping in mind that the second *cis*-coupled fluorescent protein (downstream of the IRES element) should be spectrally separated from the first one. Both mCherry and eYFP have been used as convenient tags in chimeric proteins [13,18]. Moreover, as stated above, eYFP and eCFP* on one hand, and eGFP and mCherry on the other hand, constitute two pairs of fluorescent proteins fitting the criteria of spectral separability [13,14,16]. We thus created two double color PRIGs by inserting either an open(*i.e*. without its stop codon) mCherry or an open eYFP cDNA just upstream of the BamHI site of either the pPRIGp bHA or pPRICp bHA, generating pPRIGp mChHA and pPRICp eYFPHA, respectively (Fig. 5). In these two vectors, the 5'fluorescent proteins and the 3'HA tag are in-frame but separated by the entire intact MCS. Thus, these two double color PRIGs are designed to express a mCherry -X or a eYFP-X as a chimeric protein of interest, containing or not a 3'HA tag, together with a spectrally separated fluorescent protein, either eGFP or eCFP*, respectively.

#### "Dual selection" PRIG

Finally, we wished to make possible the combination of both a drug-based selection of transduced cells and a FACS-based method to detect and quantitate the expression of the protein of interest in these cells. Thus, we further introduced an open eGFP cDNA just upstream of the BamHI site of the intact MCS of the pPRIPu vector, leading to pPRIPu eGFP vector (Fig. 5) designed to express eGFP-X chimeric proteins in transduced, puro-resistant cells.

### Versatility of the PRIG vectors

Double color PRIG vectors provide the possibility to identify transduced cells both on the basis of the IRES-controlled fluorescent proteins (thereafter referred to as XFPs), as well as by the expression of the protein of interest fused to a distinct and spectrally separable fluorescent protein. This may be valuable if the fluorescence emitted by the chimeric protein of interest is too faint (intrinsically or as a result of inadapted laser or filters) for a FACS-based sorting but sufficient (or adapted) for microscopical studies. However, the expression of the IRES-controlled XFP provides redundant information when both transduction and expression of the protein of interest can be evidenced by the signal emitted by the upstream, fused, fluorescent protein. In this case, the presence of the IRES-XFP cassette both unnecessarily increases the size of the vector and may complicate a further fluorescent characterization of the transduced cells because of the spectral overlap between XFP signal and several usual fluorochromes (e.g. eGFP/FITC). Thus, to increase the versatility of our vectors, we created the possibility to delete the IRES-XFP cassette. To this end, we inserted a site for the PacI restriction enzyme just downstream of the stop codon of the 3'HA coding sequence, and added another PacI site just upstream of the 3' LTR. Consequently, the IRES-XFP cassette is flanked by two PacI sites, not present elsewhere in PRIGs, and is thus readily and precisely removable from any of the double color PRIGs. Given that PacI is a very rare cutter, deletion of the IRES-XFP sequence will most likely be possible even when a cDNA is already cloned into these vectors. Interestingly, the PacI deletion of our two double color PRIGs generates two novel vectors which maintain spectrally separated fluorescent proteins (eYFP and mCherry), therefore remaining suitable for co-expression studies. Moreover, not only do the two double color PRIGs harbor the two PacI sites flanking the IRES-XFP cassette, but all other PRIGs designed with a « p » suffix in their name (defining the PRIG**p **sub-family, Table 1), namely the 3'HA PRIGs(pPRIGp a/b/cHA), the modular PRIGs(pPRIGpER, pPRIGp VP16HA, pPRIGp KRABHA), and the novel single color PRIGs (pPRICp aHA; pPRIYp aHA; pPRIChp aHA). Accordingly, once cloned in any of the PRIGp vectors, the chimeric and/or tagged cDNA will be both suitable for a trackable (fluorescent) transfection/transduction, as well as for a transient expression from a non-fluorescent CMV-driven vector. Finally, the PacI-deleted PRIGps, except pPRIGp KRABHA, gain another available cloning site in the MCS, HindIII, which is otherwise not unique.

**Table 1:** PRIG vector sub-series

	**PRIGps**	**Puro-resistance PRIGs**	**Previous PRIGs**
Single colour/selection	pPRIG a/b/cHA pPRICp aHA pRIYp aHA pPRIChp aHA	pPRIPu pPRIPu HAa	pPRIG pPRIG HAa/b/c
Modular	pPRIGp VP16HA pPRIGp KRABHA pPRIGp ER		
Dual colour/selection	pPRIGp mChHA pPRICp eYFPHA	PPRIPu eGFP	

### Modularity

In summary, the new PRIG vectors presented here combine the following elements: i) five 5'modules (KRAB, VP16, mCherry, eYFP, eGFP); ii) four 3' modules (HA in three reading frames and ER), all of them being designed to tag and/or to functionally modify the protein of interest; iii) and four selectable markers of transduction (eCFP*, eYFP, mCherry, PuroR) (Table 1). Clearly, we have not constructed all the possible combinations. For example, the three possible reading frames of the 3'HA tag are present only in the pPRIG background, i.e. in IRES-eGFP containing vectors. Yet, all the PRIG vectors are identical in most of their sequences, which allows easy swappings to design novel PRIGs. Moreover, the presence of the two PacI sites in most of our vectors permits the generation of new combinations even after the cloning of the cDNA of interest. This modularity may be convenient i) if a fluorescent protein distinct from eGFP is desired in PRIGs containing any regulatory module (KRAB, VP16 or ER); ii) if another pair of fluorescent proteins (e.g. mCherry and eCFP*) is desired; or iii) to obtain the correct reading frame for the 3'HA in any single color PRIGs.

### pMPI (pMigr MSCV) counterparts of the new PRIG vectors

pMigR is a retroviral vector [19] related to pPRIG since it also contains an eGFP cDNA 3' of the wild-type (« strong ») EMCV IRES sequence. However, pMigR is distinct from pPRIG in two aspects. First, it contains two LTR sequences, while pPRIG harbors a CMV promoter replacing the U3 region of the 5' LTR; pMigR is therefore less adapted than PRIGs for transient expression. Second, the LTR sequences of pMigR are of MSCV/MESV (murine stem cell virus/murine embryonic stem cell virus) origin while those of pPRIG are of PCMV (PCC4-cell passaged myeloproliferative sarcoma virus [20]) origin. Consequently, pPRIG and pMigR LTRs differ by several point mutations in the R and U5 regions, while they are identical in the U3 region. In addition, the two vectors also differ by several point mutations in the 5'part of the δgag region. pMigR vector has been commonly used to transduce pluripotent cells especially hematopoietic stem cells and embryonic stem cells [19-21]. Only four contiguous cloning sites are present in the original pMigR MCS. We designed an improved pMigR derivative, termed pMPI (previously termed pMigR-ATG [2]), which harbors some features of the PRIG vectors. Specifically, pMPI contains the MCS of PRIGs, as well as the T7 and SP6 flanking sequences, but is otherwise identical to pMigR, notably in all the viral-derived sequences (LTRs and δgag region). We also designed the pMPI HAa/b/c vectors that contain the 5'HA tag coding sequence in the three reading frames with respect to the MCS. Either pMPI or its first 5'HA derivatives have been previously used to transduce primary embryonic hematopoietic cells [22] or to transfect HEK293T (human embryonic kidney) cells [2], respectively. While designing the above described PRIG vectors, we also constructed the pMPI counteparts for some of them, namely: i) three derivatives bearing the 3' HA sequence in the three reading frames(pMPIp a/b/cHA); ii) four pMPI derivatives in which the IRES-eGFP cassette has been replaced by either the IRES-eCFP*, IRES-eYFP, IRES-mCherry or IRES-Puro (termed pMPIpeC aHA, pMPIpeY aHA, pMPIpCh aHA, pMPIPuro, respectively); and iii) a pMPIPuro derivative containing an open eGFP sequence 5' of the MCS (pMPIPu eGFP, corresponding to pPRIPu eGFP). Again, the « p » suffix indicates that the IRES-XFP cassette is flanked by two PacI sites.

### Functional tests

**Figure 6:**
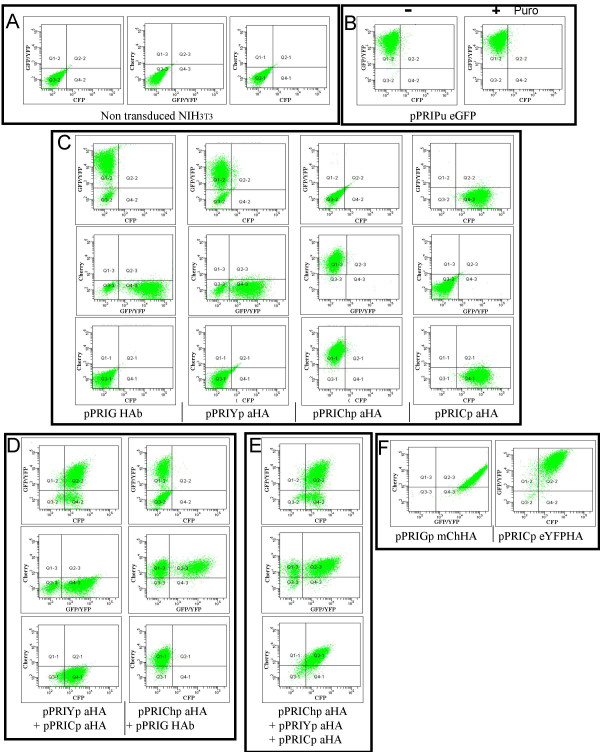
**FACS analyses of NIH3T3 cells transduced with different PRIG vectors**. Cells were analysed using a LSRII cytometer (Becton-Dickinson) 5 to 7 days after exposure to the viral supernatant(s). For each point, 10^4 ^events were recorded. A) Non-transduced NIH3T3 cells we analysed for cyan (eCFP*) yellow/green (eYFP/eGFP) or red (Cherry) fluorescences using the lasers and filters described in the *Methods *section to determine the basal level for each signal. B) Functional test of the pPRIPu eGFP vector. 48 hours after the exposure to a pPRIPu eGFP viral supernatant, cells were plated in a fresh medium containing (right panel) or not (left panel) 10 μg/ml of puromycin, and analysed for eGFP expression after 7 days of culture. All the selected and almost all the non-selected cells strongly express eGFP. Note that at this dose of puromycine, control untransduced NIH3T3 cells died in approximately 24 hours (not shown). C) Functional test of the single color vectors. Cells transduced with the indicated vector (at the bottom of each panel) were analyzed for each fluorescence. Note that under these conditions of transduction and detection, each fluorescent protein (eCFP*, eYFP/eGFP, mCherry) is mainly, if not exclusively, detected in its appropriate channel. D) Feasability of double transductions. Cells were transduced with the indicated (right hand side) mixture of two viral supernatants. In the cases of double transductions, untransduced cells, doubly transduced cells and cells transduced with either one of the two single color vectors can be discriminated from each other. E) Feasability of triple transductions. Cells were transduced with the indicated mixture of the three viral supernatants. Again, because of the minimal overlapping between the three signals, the transduction status of any cell with respect to each vector can be determined. Note however, that for D and E, the quantity of each viral supernatant was not equal in the mixture to correct for either relatively inefficient detection of the signal in our conditions (mCherry) or relatively intrinsic weakness of the protein (eCFP*) (see text). F) Functional test of the doublecolor vectors. Cells were transduced with the indicated vector (at the bottom of the panel). In each case, the vast majority of the cells are positive for the two expected signals.

**Figure 7:**
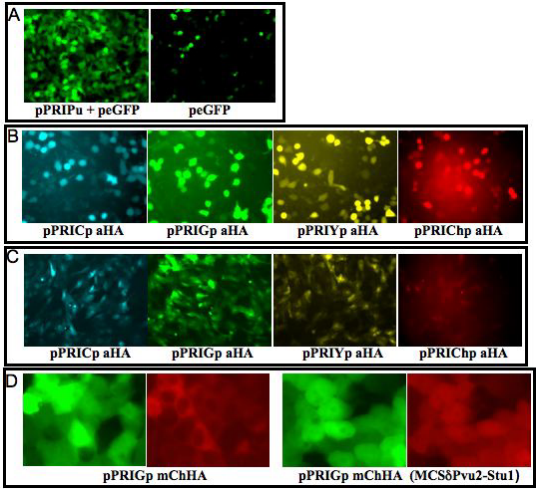
**Fluorescence microscopy analyses of cells transfected or transduced with PRIG vectors**. **A**. pPRIPu confers puro-resistance. HEK293T cells were transfected with either peGFP-N1 + pPRIPu (left) or peGFP-N1 (Clontech) + pRK5 (irrelevant carrier DNA) (right). 24 hours after transfections, cells were further grown for 30 hours in the presence of 10 μg/ml of puromycin. eGFP serves as a control for transfection efficiency. pPRIPu + peGFP-N1 transfected cells are living while most mock-transfected cells are dying. B. Single color PRIGs are expressed in HEK293T transfected cells. HEK293T cells were transfected with the indicated single color PRIG vector. Fluorescence microscopic analyses were performed 24 hours later. The signal emitted by each fluorescent protein was observed and photographed using the appropriate filter. Note that the mCherry signal is relatively weak probably due to the suboptimal excitation light delivered by our filter (546/12 nm). Moreover, a weak portion of the eGFP signal is detectable in the cyan filter, and conversely a residual portion of the eCFP* signal is detectable in the green filter. By contrast, the eYFP (green filter) and mCherry (red filter) signals are only detected in their appropriate filter. C. Single color PRIGs are expressed in transduced rat primary osteoblasts. Rat primary osteoblasts were transduced by the indicated single color PRIG vector and observed using a fluorescent microscope 48 hours later. Signals were photographed using the appropriate filter for each fluorescent protein. D. mCherry localization in HEK293T transfected cells. Left: HEK293T cells were transfected with pPRIGp mChHA. The red signal appears to be predominantly cytoplasmic, which is not observed in pPRIChp transfected cells, where mCherry shows an uniform distribution (see B). Right: HEK293T cells were transfected with a pPRIGp mChHA derivative containing an in-frame deletion of the 5' part of the MCS (PvuII/StuI). This deletion completely restores a normal (uniform) distribution of mCherry. Note that in cells transfected with pPRIGp mChHA (left) and with its PvuII/StuI deleted derivative (right), the eGFP (green, encoded by the 3' cistron) appears normally and uniformly distributed throughout the cell. The regions of interest were enlarged for a better visualization.

To validate our novel PRIGs, transfection and transduction studies were performed. First, we tested the pPRIPu eGFP derivative. To this end, mouse NIH3T3 cells were transduced with this vector, and plated 48 hours later in medium alone or in medium containing puromycin. We observed that transduced cells readily grew in medium containing up to 10 μg/ml of puromycin while control NIH3T3 cells died in approximately 24 hours under the same conditions. After 5 to 7 days, no difference was observed between transduced cells exposed to 2 or 10 μg/ml of puromycin and transduced cells grown in medium without puromycin with respect to cell viability and morphology (data not shown). This indicates that pPRIPu eGFP indeed confers resistance to puromycin. As expected, FACS analyses showed that all transduced cells exposed to puromycin express eGFP, while some cells are eGFP-negative in the population not treated with puromycin (Fig. 6A and 6B), confirming that puromycin efficiently eliminates rare untransduced cells. The same results were obtained upon transduction of NIH3T3 cells with pMPIPu eGFP (data not shown). Expression of puro-resistance and eGFP were also confirmed by fluorescence microscopy for pPRIPu eGFP transfected HEK293T cells (data not shown). Finally, the expression of the puro-resistance gene from the pPRIPu vector was verified in transiently transfected human HEK293T cells using microscopy (Fig. 7A).

Next we verified that each single color PRIG vector indeed gives the expected fluorescence in transduced cells. By FACS analyses we observed that pPRIYp aHA-, pPRICp aHA-, pPRIChp aHA- transduced cells emit yellow, blue and red fluorescence respectively, which are readily separated on a LSRII cytometer equiped with the lasers and filters described in the *Methods *section (Fig. 6C). As expected, the pPRIG HAb-transduced cells are heavily positive for eGFP expression (Fig. 6C). The expression of each single color PRIGs was also confirmed in transfected HEK293T cells and in transduced primary rat osteoblasts using fluorescence microscopy (Fig. 7B and 7C).

We next took advantage of the clear separation of each fluorescent signal in FACS analyses to demonstrate the feasability of either double (either pPRIYp aHA plus pPRICp aHA or priG-HAb plus pPRIChp aHA) or triple (pPRIYp aHA plus pPRICp aHA plus pPRIChp aHA) co-transductions. These experiments show that cells exposed to the mixtures of viral supernatants can be characterized for each signal whose origin is unambigously ascribed to one of the fluorescent proteins (Fig. 6D and 6E). Thus, single color PRIGs are potentially suitable to simultaneously express up to three genes in transduced cells, with the possibility to identify the subpopulations expressing any of them or any combination (including all) of them. For all these experiments, similar results were obtained using the pMPI counterparts of these single color PRIGs (data not shown). Moreover, pPRIPu can be used together with any of the single color PRIGs, further extending the possibilities of multiple transductions. Again, the same results were obtained upon transduction with the pPMI counterparts of the single color PRIGs, namely pMPIpeC aHA, pMPIpeY aHA, pMPIpCh aHA, and MPI HAa (data not shown). It is worth noting, however, that in order to improve the balance between the different fluorescent signals, we had to correct for the relatively weak eCFP* and mCherry fluorescences compared to eYFP or eGFP, by exposing the cells to greater amounts of pPRICp aHA or pPRIChp aHA viral supernatants (500 μl instead of 50 μl for pPRIYp aHA or pPRIG HAb (Fig. 6D and 6E)). The difference in fluorescence intensities is probably due to either the intrinsically low brightness of eCFP* compared to eGFP or eYFP, or to the non-optimal excitation of mCherry by our 488 nm laser [13,15]. Indeed, when translated from the same mRNA in double color PRIG transduced cells, the fluorescent signals exhibit similar differential intensities (see below) ruling out that the differences observed among single color PRIG-transduced cells stem from differential viral titer.

Finally, we show that the vast majority of NIH3T3 cells transduced with one of the double color PRIGs (either pPRIGp mChHA or pPRICp eYFPHA) is positive for the two expected fluorescences (Fig. 6F). In these cases, it is impossible to experimentally balance the fluorescent signals emitted by the proteins of each couple, since the two proteins are coded by a single vector. Consequently, though most cells are doubly positive, eGFP appears stronger than mCherry upon transduction with pPRIGp mChHA and eYFP appears stronger than eCFP* upon transduction with pPRICp eYFPHA.

During the course of these experiments, we also observed that mouse NIH3T3 fibroblasts or rat osteoblasts transduced with pPRIGp mChHA display a predominant cytoplasmic localization of the red signal ("pitted mCherry "), while eGFP exhibit a normal, uniform, distribution (data not shown). This unexpected mCherry localization is also observed in HEK293T cells transfected with this vector (Fig. 7D, left), but not in pPRIpCh aHA-transduced or transfected cells (Fig. 7B and data not shown), which display an uniform mCherry localization. We strongly suspect that the disturbed localization of mCherry originating from the pPRIGp mChHA is caused by its fusion to the MCS. Indeed, in one reading frame, the MCS encodes a short peptide in its 5' part which somehow disturbs the localization of mCherry. Accordingly the same effect, although less pronounced, is observed for the 5' fluorescent proteins of pPRICp eYFPHA and for pPRIPu eGFP which fuse eYFP or eGFP, respectively, to the same reading frame of the MCS (data not shown). Moreover, the insertion of mCherry 5' of the MCS in the two other reading frames leads to an uniform mCherry distribution. Finally, mCherry expressed from the pPRIGp mChHA recovers a normal homogeneous localization upon the in-frame PvuII/StuI deletion of the 5' part of the MCS (or upon the almost entire MCS in-frame XhoI/SalI deletion), but not upon the in-frame ClaI/AsuII deletion (Fig. 7D, right and data not shown). Thus, users may want to delete the 5' part of the MCS (between the PvuII site and the ClaI site and encoding the sequence LEFSRSLCI) upon cloning when this reading frame is in phase with their cDNA.

## Conclusion

We describe here a new generation of PRIG vectors. They keep the original improvements of the pPRIG vector with respect to its ancestor pAP2, *i.e*. a far more complete and convenient MCS and a wild type strong EMCV IRES element, and provide several modifications to extend their use to a wide variety of experimental settings or biological issues, as detailed above. Moreover, we introduced some other changes to enhance their versatility, and duplicated eight of them in a pMigR (MSCV) background for their use in stem cells. All constructs were generated by restriction and modification enzymes and all cloning modifications were verified by sequencing. In addition, the viral parts (*i.e*. from the 5' end of the 5' LTR to the 3' end of the 3' LTR) of the ancestor pAP2 vector and of two of its final descendents (pPRIGp aHA and pPRIYp aHA) were entirely sequenced and show no discrepancy with respect to the expected sequence. Finally, their reliability was further demonstrated by the experimental validations we show here. All these vectors are made freely available to the scientific community and all the maps and sequences will be posted on our website in the near future [23].

## Methods

### Constructs

All the DNA constructs were made using standard procedures. Restriction and modification enzymes were purchased from Fermentas, New England Biolabs and Invitrogen. Plasmids were prepared using CsCl procedure or Purelink Midiprep kits (Invitrogen). The VP16 sequence was taken from the pVP16 vector (Clontech, a gift from R. Galien). It is worth mentioning that this vector contains the « full-length » (aa 410 to 490 of the HSV1 protein) VP16 acidic transactivation domain (plus an N-terminal nuclear targeting domain) and not the 411–455 subdomain as described by the supplier. The KRAB domain of the human KOX1 protein was isolated from a PAX3-KRAB encoding vector ([11], a gift from Drs Rauscher and Hongzuang, Wistar Institute, Philadelphia, USA). eGFP and eYFP cDNAs were isolated from the peGFP-C1 and peYFP-C1, respectively (Clontech). mCherry [13] cDNA was a gift from Dr Tsien (San Diego, USA). The sequence encoding the ER hormone binding domain containing the G525R mutation was provided by D. Monté [4]. The eCFP cDNA bearing the H148D mutation (eCFP*) was a gift from Dr Graihle (Pasteur Institute, Seoul, South Korea).

### Verification of the constructs

Detailed cloning strategies, maps and sequences of all vectors are available upon request, but some points should be underlined. First, we did not use PCR. All of our constructs were built using restriction and modification enzymes. Second, we have extensively sequenced all the constructs presented here. All the viral portions of pAP2 and pMigR plasmids have been sequenced and several differences were found compared to the theoretical sequence, and at least all the module and/or fluorescent/selectable proteins in each construct have also been sequenced. In summary, we are confident that the entire nucleotide sequence of each construct posted on the web site [23] is error free.

Third, eGFP cDNA from pMigr/pMPI vectors differs from that of pAP2 and of its first descendents pPRIG and pPRIG HA a/b/c in two nucleotides and one amino acid (V164 in pAP2 type eGFP, A in pMigr/pMPI-type, if the first Met residue is 1). pPAP2-type and pMigr/pMPI-type eGFPs however exhibit indistinguishable fluorescent properties. Due to the phylogeny of our new PRIG vectors (reflecting the cloning strategy designed to introduce the distal PacI site), all the herein described pPRIG vectors (as well as all pMPI vectors) contain the pMigr/pMPI-type eGFP, except the pPRIPu eGFP whose 5'eGFP originates from peGFP-C1 which is identical to the pAP2-type eGFP.

Fourth, we previously reported that the efficiency of the EMCV IRES is strongly dependent upon the integrity of its last ATG, which should be used as start codon for the downstream eGFP cDNA. Thus, in each construct where eGFP was replaced by another cDNA (eCFP*, eYFP, mCherry, Puro-R), we kept the last IRES ATG intact and in-frame with the natural start codon of the cDNA. The high efficiency of the IRES in all our constructs is confirmed by strong expression of the fluorescent proteins or Puro-resistance of transduced cells (see text).

### Transfection, transduction, FACS and immunofluorescence analyses

HEK293T (a HEK293 subline containing the simian virus 40 (SV40) T antigen) were cultured in DMEM medium supplemented with 10% FCS and antibiotics, mouse NIH3T3 fibroblasts in a mixture of 3/4 of DMEM and 1/4 of F12 medium (Invitrogen) supplemented with 10% FCS and antibiotics. Primary rat osteoblasts were obtained and grown according to ref [24].

For NIH3T3 cells, all the retroviral supernatants were prepared by transfecting human 5 × 10^5 ^HEK293T in 6 well dishes cells by 0.4 μg of pCMV-intron gag-pol, 0.4 μg of the FB-Mo-Salf vector encoding the ecotropic MLV env protein (two gifts from F.L. Cosset, CNRS Lyon, France [25]) together with 0.8 μg of the PRIG, pMPI or pMPI derivatives using Fugene 6 (Roche) as transfection reagent. 24 hours later, the indicated volume of viral supernatant(s) were filtered on 0.45 μm filters and added to 10^5 ^NIH3T3 mouse fibroblasts in the presence of 5 μg/ml of polybrene. 5 to 7 days later, NIH3T3 cells were analysed for the expression of the fluorescent protein through FACS analyses using LSRII (Becton Dickinson). eCFP* (eCFP containing the H148D substitution) was excited by a 405 nm laser and its emission was collected by a 440/40 nm filter. eGFP, eYFP and mCherry were excited by a 488 nm laser, emission of both eGFP and eYFP was collected by a 510/20 nm filter while mCherry emission was collected by a 610/20 nm filter. 10^4 ^events were recorded.

For HEK293T cells and rat primary osteoblasts, transfections and transductions were performed according to the protocol described in ref [2]. Fluorescence microscopy was performed using a Axiovert 40 CFL microscope (Zeiss) at 20× magnification. The eGFP and eYFP signals were observed using the filterset 44 (Zeiss; excitation: 475/40; emission: 530/50; beamsplitter: 455, "green filter"), mCherry signal using the Filterset 15 (Zeiss; excitation: 546/12; emission: LP 590; Beamsplitter: 580, "red filter") and eCFP* signal was observed using the HE 47 filter (Zeiss; excitation: 436/25; emission: 480/40; Beamsplitter: 455, "cyan filter").

## Abbreviations

eCFP/eGFP/eYFP: enhanced cyan/green/yellow fluorescent protein. IRES: Internal Ribosome Entry Site. LTR: Long Terminal Repeat. ECMV: encephalomyocarditis virus. MSCV: murine stem cell virus. PCMV: PCC4-cell passaged myeloproliferative sarcoma virus. ER: hormone binding domain of the mouse estrogen receptor alpha. VP16: viral protein 16. KRAB: krüppel-associated box. Puro-R : puromycine resistance gene. FACS : fluorescence-activated cell-sorting.

## Authors' contributions

PM and OAC designed and constructed the vectors. OAC wrote the paper, PM helped in the writing of the paper. PM made the figures with the help of OA. OAC and YL performed the FACS analyses, including NIH3T3 cell transduction. PM, PP and KEB performed the fluorescence analyses, including rat osteoblast transduction and HEK293T cell transfection. KEB edited the manuscript. All authors read and approved the final manuscript.

## References

[B1] Galipeau J, Li H, Paquin A, Sicilia F, Karpati G, Nalbantoglu J (1999). Vesicular stomatitis virus G pseudotyped retrovector mediates effective in vivo suicide gene delivery in experimental brain cancer. Cancer Res.

[B2] Martin P, Albagli O, Poggi MC, Boulukos KE, Pognonec P (2006). Development of a new bicistronic vector with strong IRES activity. BMC Biotechnol.

[B3] Eilers M, Picard D, Yamamoto KR, Bishop JM (1989). Chimaeras of myc oncoprotein and steroid receptors cause hormone-dependent transformation of cells. Nature.

[B4] Littlewood TD, Hancock DC, Danielan PS, Parker MG, Evan GI (1995). A modified oestrogen receptor ligand-binding domain as an improved switch for the regulation of heterologous proteins. Nucleic Acids Res.

[B5] Berry M, Metzger D, Chambon P (1990). Role of the two activating domains of the oestrogen receptor in the cell-type and promoter context dependent agonistic activity of the anti-oestrogen 4-hydroxytamoxifen. EMBO J.

[B6] Zeisig BB, Schreiner S, Garcia-Cuellar MP, Slany RK (2003). Transcriptional activation is a key function encoded by MLL fusion partner. Leukemia.

[B7] Steiner AB, Engleka MJ, Lu Q, Piwarzyk EC, Yaklichkin S, Lefebvre JL, Walters JW, Pineda-Salgado L, Labosky PA, Kessler DS (2006). FoxD3 regulation in the Speemann organizer is essential for Xenopus dorsal mesoderm development. Development.

[B8] Regier JL, Shen F, Triezenberg SJ (1993). Pattern of aromatic and hydrophobicamino acids critical for one of two subdomains of the VP16 transcriptional activator. Proc Natl Acad Sci USA.

[B9] Margolin JF, Friedman JR, Meyer WK, Vissing H, Thiesen HJ, Rauscher FJ (1994). Krüppel-associated boxes are potent transcriptional repression domains. Proc Ntl Acad Sci USA.

[B10] Deuschle U, Meyer WK, Thiesen HJ (1995). Tetracycline-reversible silencing of eukaryotic promoters. Mol Cell Biol.

[B11] Fredericks WJ, Kasirajan A, Herlyn M, Friedman JR, Rauscher FJ (2000). Mol An engineered PAX3-KRAB transcriptional repressor inhibits the malignant phenotype of alveolar rhabdomyosarcoma cells harboring the endogenous PAX3-FKHR oncogene. Mol Cell Biol.

[B12] Sanchez JP, Ullman C, Moore M, Choo Y, Chua NH (2006). Regulation of Arabidopsis thaliana 4-coumarate:coenzyme-A ligase-1 expression by artificial zinc finger chimeras. Plant Biotechnol J.

[B13] Shaner NC, Campbell RE, Steinbach PA, Giepmans BNG, Palmer AE, Tsien RY (2004). Improved monomeric red, orange and yellow fluorescent proteins derived from Discosoma sp. Red fluorescent protein. Nature Biotech.

[B14] Shaner NC, Steinbach PA, Tsien RY (2005). A guide to choosing fluorescent proteins. Nature Methods.

[B15] Rizzo MA, Springer GH, Granada B, Piston DW (2004). An improved cyan fluorescent protein variant useful for FRET. Nature Biotech.

[B16] He Liusheng, Wu Xiaoli, Simone James, Hewgill Derek, Lipsky PeterE (2005). Determination of tumor necrosis factor receptor-associated factor trimerization in living cells by CFP→YFP→mRFP FRET detected by flow cytometry. Nucleic Acids Res.

[B17] Takahashi K, Yamanaka S Induction of pluripotent stem cells from mouse embryonic and adult fibroblast cultures by defined factors. Cell.

[B18] Vessey JP, Vaccani A, Xie Y, Dahm R, Karra D, Kiebler MA, Macchi P (2006). Dendritic localization of the translational repressor Pumilio 2 and its contribution to dendritic stress granules. J Neurosci.

[B19] Pear WS, Miller JP, Xu L, Pui JC, Soffer B, Quackenbush RC, Pendergast AM, Bronson R, Aster JC, Scott ML, Baltimore D (1998). Efficient and rapid induction of a chronic myelogenous leukemia-like myeloproliferative disease in mice receiving P210 bcr/abl-transduced bone marrow. Blood.

[B20] Cherry SR, Biniszkiewicz D, van Parijs L, Baltimore D, Jaenisch R (2000). Retroviral expression in embryonic stem cells and hematopoietic stem cells. Mol Cell Biol.

[B21] Grez M, Akgün E, Hilberg F, Ostertag W (1990). Embryonic stem cell virus, a recombinant murine retrovirus with expression in embryonic stem cells. Proc Ntl Acad Sci USA.

[B22] Giroux SJ, Alves-Leiva C, Lécluse Y, Martin P, Albagli O, Godin I (2007). Gene transfer to pre-hematopoietic and committed hematopoietic precursors in the early mouse yolk sac: a comparative study between in situ electroporation and retroviral transduction. BMC Dev Biol.

[B23] http://www.unice.fr/FRE3094/PRIG/PRIG_home.html.

[B24] Morvan Frederic, Boulukos Kim, Clément-Lacroix Philippe, Roman Sergio Roman, Suc-Royer Isabelle, Vayssière Béatrice, Ammann Patrick, Martin Patrick, Pinho Sonia, Pognonec Philippe, Mollat Patrick, Niehrs Christof, Baron Roland, Rawadi Georges (2006). Deletion of a single allele of the Dkk1 gene leads to an increase in bone formation and bone mass. J Bone Miner Res.

[B25] Lavillette D, Maurice M, Roche C, Russell SJ, Sitbon M, Cosset FL (1998). A proline-rich motif downstream of the receptor binding domain modulates conformation and fusogenicity of murine retroviral envelopes. J Virol.

